# Conservative Management of Bowel Injury Following Renal Cryoablation: A Series of Five Cases

**DOI:** 10.7759/cureus.96163

**Published:** 2025-11-05

**Authors:** Masataka Kubo, Kosuke Iwatani, Takahiro Higuchi, Takahiro Kimura, Jun Miki

**Affiliations:** 1 Department of Urology, The Jikei University School of Medicine, Kashiwa Hospital, Chiba, JPN; 2 Department of Radiology, The Jikei University School of Medicine, Kashiwa Hospital, Chiba, JPN; 3 Department of Urology, The Jikei University School of Medicine, Tokyo, JPN

**Keywords:** bowel injury, complication, conservative treatment, cryoablation, renal cell carcinoma

## Abstract

Percutaneous cryoablation (PCA) is a minimally invasive treatment for small renal tumors, particularly in older or high-risk patients. Although rare, bowel injury is a recognized complication with the potential for severe outcomes. We report five cases (1.4%) of bowel injury among 363 PCA procedures performed between 2015 and 2023 at our institution. All injuries were confined to the retroperitoneum and managed conservatively without surgery. Mechanisms included ischemia, thermal injury, mechanical puncture, patient movement, and delayed abscess formation. Representative cases included a colorenal fistula that closed spontaneously and a renal abscess requiring drainage. Despite varied mechanisms, all patients were clinically stable with localized findings. Conservative treatment with antibiotics, fasting, and imaging follow-up was effective. No patients developed peritonitis or required surgical intervention. Conservative management may be appropriate for certain patients with bowel injuries after renal PCA, when the clinical condition is stable and the injury is anatomically confined.

## Introduction

Percutaneous cryoablation (PCA) is an established treatment for small renal tumors, especially in older patients and those with comorbidities who are poor candidates for surgery [1-3]. The technique allows real-time visualization of the iceball under imaging, which helps to target the tumor precisely and minimize damage to adjacent structures [4,5].

At our institution, PCA is performed under computed tomography (CT) guidance. In some cases, transcatheter arterial embolization (TAE) and lipiodol marking are used to improve visualization. Hydrodissection is routinely used to separate the bowel and other organs from the ablation zone.

Bowel injury is a recognized but rare complication of PCA, with an incidence of 0.3%-2.2% [6-9]. When it occurs, it can cause serious complications. Most previous reports describe surgical repair in cases with perforation, peritonitis, or systemic infection [10]. More recently, some authors have suggested that conservative management may be possible in carefully selected patients [6,7,11,12].

Here we describe five patients with bowel injury after PCA for renal tumors. All were successfully managed without surgery. Two representative cases are presented in detail, and the remaining three are summarized.

## Case presentation

In total, 363 PCA procedures for renal tumors were performed at our institution, The Jikei University School of Medicine, Kashiwa Hospital, Chiba, Japan, between 2015 and 2023. Five patients (1.4%) developed bowel injury. All cases were confined to the retroperitoneum and were treated conservatively. Table 1 summarizes the clinical characteristics and outcomes.

**Table 1:** Summary of five cases This is a summary of the cases. Gastrointestinal tract injury or fistula was observed in all cases; however, most complications were mild, classified as Clavien-Dindo Grade I [14,15]. R.E.N.A.L Score: The R.E.N.A.L. Nephrometry Score; R: radius (maximal tumor diameter); E: exophytic/endophytic properties of the tumor; N: Nearness of the deepest portion of the tumor to the collecting system or renal sinus; A: anterior (a)/posterior (p) descriptor; L: location relative to the polar line [13]. Clavien-Dindo Grade definitions are as follows: Grade I: any deviation from the normal postoperative course without the need for pharmacological treatment or surgical, endoscopic, and radiological interventions. Grade II: requiring pharmacological treatment with drugs other than such allowed for grade I complications. Grade III: requiring surgical, endoscopic, or radiological intervention. Grade IIIa: intervention not under general anesthesia. Grade IIIb: intervention under general anesthesia. Grade IV: life-threatening complication requiring ICU management. Grade IVa: single organ dysfunction. Grade IVb: multiorgan dysfunction. Grade V: death of a patient [14,15]. RCC: renal cell carcinoma; ccRCC: clear cell renal cell carcinoma; TAE: transcatheter arterial embolization

Case	Gender	Age (years)	Reasons for Surgical Infeasibility	Biopsy	RCC type	Tumor location	Tumor Size (mm)	R.E.N.A.L score [13]	TAE	Hydrodissection	Postion	Event	Clavien–Dindo grade [14,15]	Symptoms	Treatment
1	Male	84	Complications	Yes	ccRCC	Light/Middle	38	6a	Yes	Yes	Right lateral	Colorenal fistula	Ⅰ	None	Fasting for one month
2	Male	70	Complications	Yes	Papillary RCC	Light/Middle	52	9a	Yes	Yes	Right lateral	Colorenal fistula, Renal abscess	Ⅲa	Fever, flank pain one month later	CT-guided percutaneous drainage
3	Male	83	Complications	Yes	ccRCC	Right/Middle	39	9a	Yes	Yes	Left lateral	Duodeorenal fistula	Ⅰ	None	None
4	Female	82	Complications	None	Unknown	Right/Lower pole	53	6x	Yes	Yes	Left lateral	Ascending colon injury	Ⅰ	None	Fasting for five days
5	Female	45	Bilateral multiple tumors, complications	None	Unknown	Right/Lower pole	30	5x	Yes	Yes	Left lateral	Descending colon injury	Ⅰ	None	None

Case 1 

**Figure 1:**
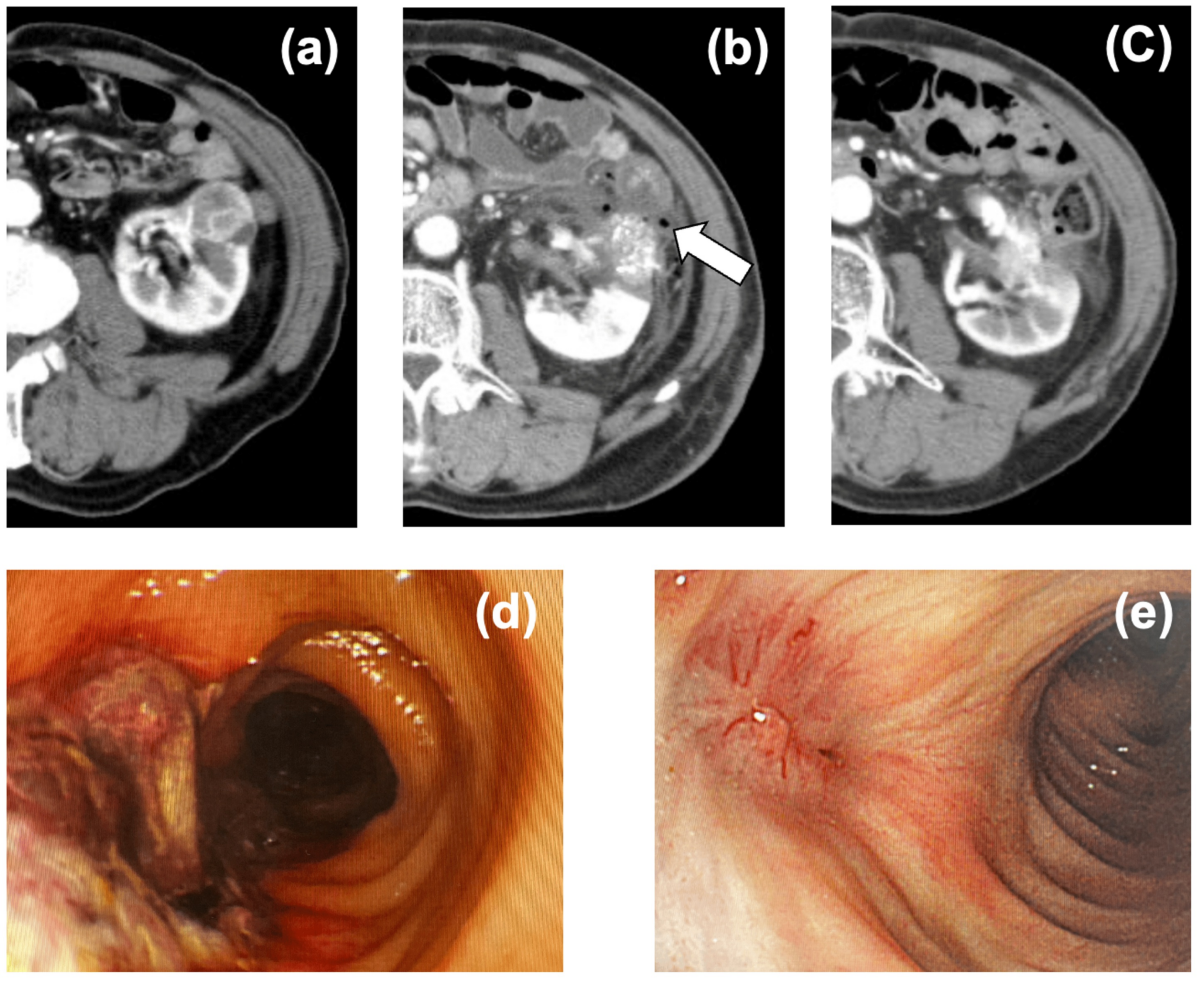
Case 1: Colorenal fistula following PCA (a) Contrast-enhanced CT showing a 38 mm exophytic renal cell carcinoma in the left kidney. (b) Post-procedural CT demonstrating an ischemic area of the colonic wall (arrow). (c) Follow-up CT four weeks later showing an improvement in the enhancement of the colonic wall. (d-e) Colonoscopy images demonstrating bowel wall ulceration at onset (d) and resolution after treatment (e). PCA: percutaneous cryoablation

An 84-year-old man underwent CT-guided PCA for a 38 mm left exophytic renal cell carcinoma (RCC). The procedure was technically successful. A CT on the following day revealed reduced mural enhancement of the descending colon (Figure 1). A colonoscopy showed an 8 cm mucosal ulceration without perforation, resembling ischemic colitis. The ulcer was larger than the iceball, suggesting ischemic change rather than direct thermal injury.

The patient remained stable but later developed pneumaturia. Two weeks after PCA, CT demonstrated a colorenal fistula. A barium enema at two months showed a diverticulum-like outpouching without leakage. Colonoscopy at three months confirmed spontaneous closure. He recovered completely with fasting and antibiotics, and surgery was not required.

Case 2 

**Figure 2:**
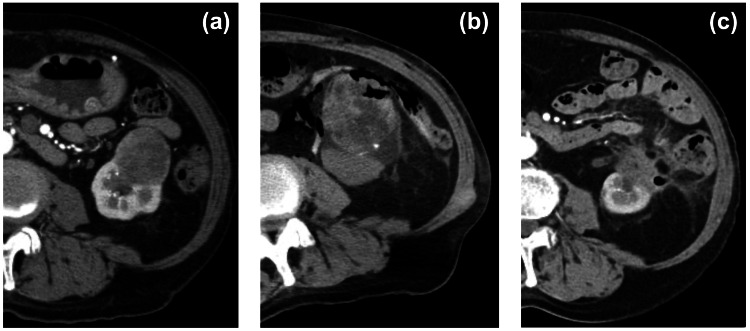
Case 2: Delayed renal abscess associated with bowel injury (a) Contrast-enhanced CT showing a 42 mm papillary renal cell carcinoma in the right kidney. (b) CT obtained four weeks later revealing a renal abscess adjacent to the ablation zone. (c) Follow-up CT obtained three months later showing improvement of the renal abscess.

A 70-year-old man with a 42 mm papillary RCC underwent embolization followed by PCA. The initial course was uneventful, and he was discharged on the following day. One month later, he presented with fever and flank pain. CT showed a renal abscess near the ablation zone (Figure 2). CT-guided drainage and broad-spectrum antibiotics were started. Because of poor cardiac function, surgery was contraindicated. The abscess improved, and the catheter was removed after two weeks.

The abscess recurred one month later and was treated again with drainage and antibiotics. Both episodes resolved without laparotomy. He ultimately recovered completely, and follow-up imaging showed no residual abscess or tumour recurrence. This case illustrates a delayed bowel-related complication and the importance of long-term vigilance after PCA.

Three other cases 

Case 3

**Figure 3:**
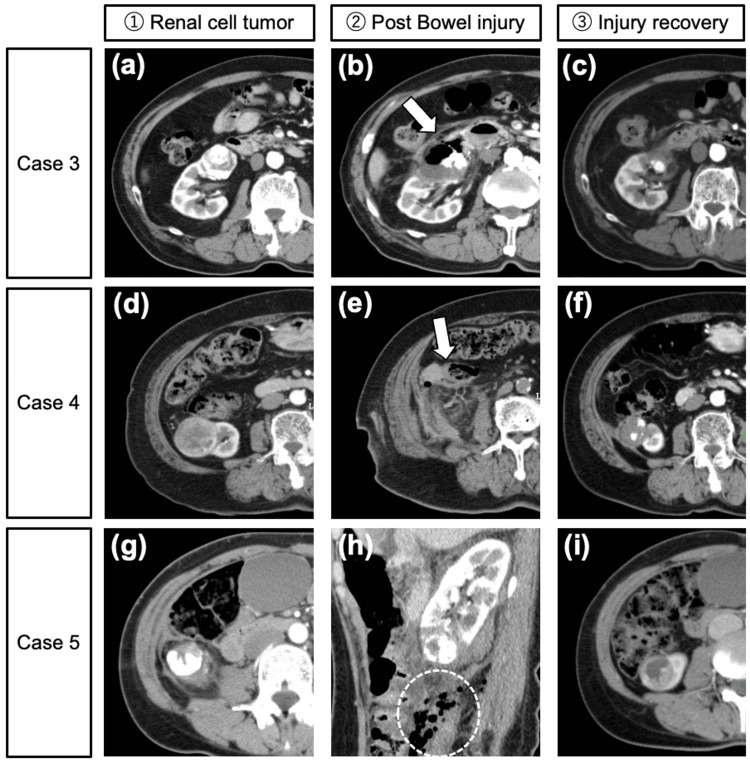
Cases 3-5: Bowel injuries following PCA Case 1: Duodenal Fistula (a) Preoperative image showing a 39 mm ccRCC in the right kidney. (b) Post-PCA CT image demonstrating a duodenal fistula (arrow). (c) The fistula spontaneously closed without the need for intervention. Case 2: Ascending colon injury (d) Preoperative image showing a 53 mm mass in the right kidney. (e) CT image demonstrating thickening of the colon wall (arrow) suspected to be due to needle injury during hydrodissection. (f) The complication spontaneously resolved with bowel rest. Case 3: Descending colon injury (g) Preoperative image showing a 30 mm mass in the right kidney. (h) Post-PCA CT image showing perinephric and colonic air (dotted circle). (i) Spontaneous resolution with conservative management. PCA: percutaneous cryoablation

An 83-year-old man developed a duodenorenal fistula one month after PCA. He remained asymptomatic, and imaging confirmed spontaneous closure (Figures 3a-3c).

Case 4

An 82-year-old woman developed localized thickening of the colon wall, likely due to needle puncture during hydrodissection (Figures 3d-3f). She was asymptomatic and recovered with fasting and antibiotics.

Case 5

A 45-year-old woman with von Hippel-Lindau syndrome developed perinephric air and mild bowel hypoenhancement after involuntary movement during PCA (Figures 3g-3i). Conservative observation led to complete resolution.

## Discussion

Bowel injury is an uncommon but important complication of PCA. In our series, five injuries occurred in 363 procedures (1.4%), consistent with previous reports [6-9]. All patients were treated conservatively and recovered without surgery.

The mechanisms differed among cases: direct thermal damage, ischemic necrosis, mechanical puncture during hydrodissection, insufficient bowel displacement due to patient movement, and delayed infection with abscess. Despite these differences, all injuries were retroperitoneally confined. None of the patients had intraperitoneal free air, diffuse peritonitis, or unstable vital signs. Symptoms were mild or absent in most patients. These features likely explain the favorable outcomes with conservative management.

As summarized in Table 2, previous studies have described both conservatively managed cases and severe complications requiring surgery (Clavien-Dindo grade IIIb) [6,7,10-12,14-16]. Surgical cases usually present with severe symptoms such as hematochezia, syncope, or deep ulceration. By contrast, our patients had mild or no symptoms and only localized findings. These differences suggest that symptom severity, systemic condition, and anatomical extent are critical in deciding between conservative and surgical management. In stable patients without systemic infection or intraperitoneal perforation, a conservative approach appears reasonable.

**Table 2:** Previous studies Previous cases are summarized, detailing patient demographics, tumor characteristics, and management strategies. The most common complications included colorectal fistula and a high frequency of severe complications (Clavien-Dindo grade ≥III) [14,15]. R.E.N.A.L Score: The R.E.N.A.L. Nephrometry Score; R: radius (maximal tumor diameter); E: exophytic/endophytic properties of the tumor; N: Nearness of the deepest portion of the tumor to the collecting system or renal sinus; A: anterior (a)/posterior (p) descriptor; L: location relative to the polar line [13].

Case	Author	Gender	Age (years)	Tumor size (mm)	R.E.N.A.L score [13]	Tumor location	Event	Clavien–Dindo grade [14,15]	Symptoms	Cause	Surgical intervention	Treatment
1	Hussein et al. [6^]^	Unknown	Unknown	None	None	None	Colorenal fistula	Ⅲ	None	Hydrodissection techniques	No	CT-guided drainage
2	Nicholas et al. [7]	Unknown	Unknown	43	5a	Left lower pole	Colonic microperforation	≦Ⅱ	Hematochezia, abdominal pain	None	No	Conservative
3	Nicholas et al. [7]	Unknown	Unknown	43	8a	Right lower pole	Duodenal and ureteral injury	Ⅲa	Abdominal pain	None	No	Abscess drainage
4	Gobara et al. [10]	Male	87	None	None	Left	Deep ulceration	Ⅲb	Left upper quadrant tenderness	None	Yes	Partial colectomy
5	Miyazaki et al. [11]	Male	50	47	4a	Right upper pole	Tumor-bowel fistula	Ⅲa	Right upper quadrant tenderness	Large tumor size after right hemicolectomy	No	Ileus tube insertion
6	Morgan et al. [12]	Male	62	None	None	Left mid-pole and posterior upper pole	Colorenal fistula	Ⅱ	Pneumaturia and left flank pain	None	No	Antibiotics
7	John et al. [13]	Female	76	45	None	Left	Colorenal fistula	Ⅲb	Hematochezia, syncope, abdominal pain	Possibly inadvertently puncture the bowel	Yes	Left nephrectomy, left colectomy

Preventive measures are essential. Hydrodissection is the standard method to separate the bowel from the ablation zone, but it can fail if the injected fluid is insufficient, if the patient moves due to inadequate analgesia, or if the needle position is not optimal. Careful planning, effective pain control, and close monitoring via imaging during ablation are required. Even with preventive strategies, delayed complications may occur, as in our patient who developed an abscess one month later. This highlights the need for long-term follow-up.

This study had several limitations. It was a retrospective analysis from a single center, and the number of cases was small. Many of our patients were older or had significant comorbidities, which made surgical intervention less desirable. In such situations, conservative management is often the most appropriate option. Because bowel injury after PCA is rare, organizing a prospective multicenter trial would be challenging. Instead, it is important to accumulate further real-world data to refine patient selection and identify predictors of favorable outcomes.

## Conclusions

Bowel injury following renal PCA is rare but clinically important. Crucially, all five identified cases were successfully treated with conservative management, avoiding the morbidity of surgery. This strategy is a safe and viable option for clinically stable patients with injuries confined to the retroperitoneum. Moving forward, continued emphasis must be placed on meticulous preventive techniques and rigorous case selection to minimize risk. Furthermore, future studies should focus on establishing standardized long-term follow-up protocols for conservatively managed injuries.
